# Establishing Preferred Product Characterization for the Evaluation of RNA Vaccine Antigens

**DOI:** 10.3390/vaccines7040131

**Published:** 2019-09-27

**Authors:** Cristina Poveda, Amadeo B. Biter, Maria Elena Bottazzi, Ulrich Strych

**Affiliations:** 1 Department of Pediatrics, National School of Tropical Medicine, Baylor College of Medicine, One Baylor Plaza, BCM113 Houston, TX 77030, USA; Cristina.Poveda@bcm.edu (C.P.); Amadeo.Biter@bcm.edu (A.B.B.); 2 Texas Children’s Hospital Center for Vaccine Development, Baylor College of Medicine, 1102 Bates Street, Houston, TX 77030, USA; bottazzi@bcm.edu; 3 Department of Pediatrics and Molecular Virology & Microbiology, National School of Tropical Medicine, Baylor College of Medicine, One Baylor Plaza, BCM113 Houston, TX 77030, USA; 4 Department of Biology, College of Arts and Sciences, Baylor University, Waco, TX 76798, USA

**Keywords:** product characteristics, capping, dendritic cells, antigen presenting cells, therapeutics

## Abstract

The preferred product characteristics (for chemistry, control, and manufacture), in addition to safety and efficacy, are quintessential requirements for any successful therapeutic. Messenger RNA vaccines constitute a relatively new alternative to traditional vaccine development platforms, and thus there is less clarity regarding the criteria needed to ensure regulatory compliance and acceptance. Generally, to identify the ideal product characteristics, a series of assays needs to be developed, qualified and ultimately validated to determine the integrity, purity, stability, and reproducibility of a vaccine target. Here, using the available literature, we provide a summary of the array of biophysical and biochemical assays currently used in the field to characterize mRNA vaccine antigen candidates. Moreover, we review various in vitro functional cell-based assays that have been employed to facilitate the early assessment of the biological activity of these molecules, including the predictive immune response triggered in the host cell. Messenger RNA vaccines can be produced rapidly and at large scale, and thus will particularly benefit from well-defined and well-characterized assays ultimately to be used for in-process, release and stability-indications, which will allow equally rapid screening of immunogenicity, efficacy, and safety without the need to conduct often lengthy and costly in vivo experiments.

## 1. Introduction

Over the last decade, messenger RNA (mRNA) has emerged as a new platform technology for the development of vaccines not just for anti-cancer therapeutics but also against infectious diseases. As reviewed in the accompanying manuscript by Versteeg et al., there is now even progress in the challenging development of mRNA vaccines against parasitic infections where a low-cost and high-throughput vaccine development platform is urgently needed [1].

Messenger RNA vaccines are composed of one or more nucleic acids encoding for a single or multiple vaccine antigen candidates, formulated in a delivery vehicle and then directly delivered into the host cell. These delivery vehicles include lipid- and protein-based molecules, as well as various types of polymeric scaffolding [2,3,4,5,6,7,8,9].

The mRNAs’ biochemical and biophysical properties promise to allow for simple multiplexing of vaccine antigens without the formulation complications that can be encountered with protein-based vaccines, or the potential risks from attenuated whole-cell vaccines. Moreover, in contrast to DNA vaccines, RNA vaccines exert their function directly in the cytoplasm, depending on neither transport mechanisms nor on the cellular transcription machinery. The fact that RNA cannot integrate into the host genome and that it has a short half-life that limits its persistence in the body contributes to a favorable prediction of its safety profile. A longer half-life can be achieved with self-amplifying mRNA (SAM); SAM vaccines are engineered into the backbone of an RNA virus genome, allowing amplification of the antigen-coding region resulting in higher levels of expression. Since the virus’ structural genes are absent in SAM constructs, there is no associated risk of infection [10,11].

As reviewed elsewhere [1,6], mRNA turns cells into antigen production factories; as vaccines, these antigens trigger potent cellular and humoral immune responses.

**Figure 1:**
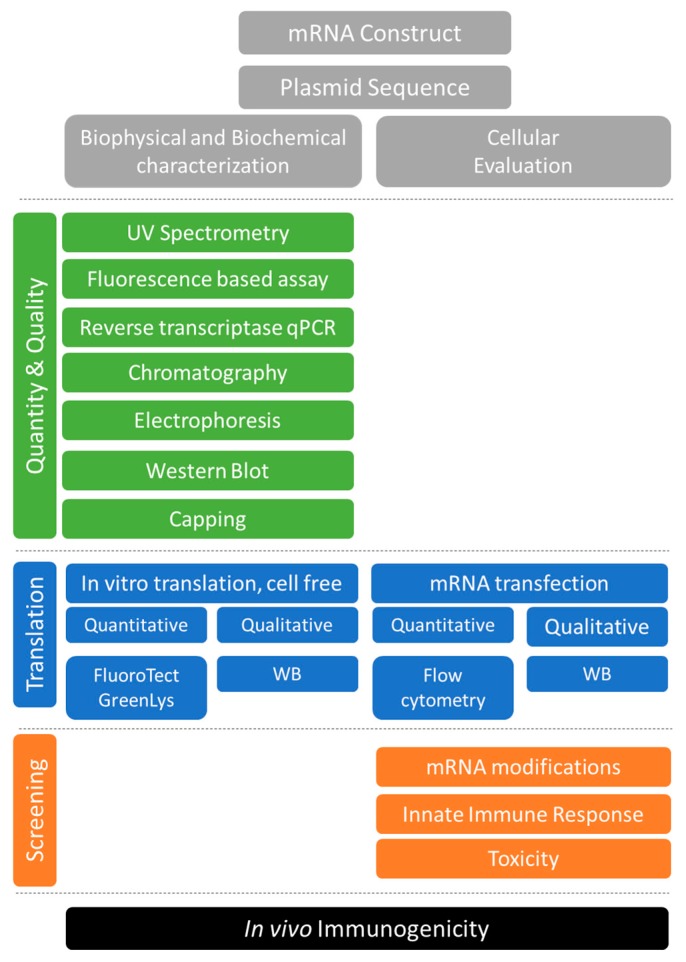
Tools for the quality control and cell-based functional evaluation of mRNA vaccine antigens.

Long-established for other biologics, an array of quality control measures (for chemistry, control, and manufacture) will need to be developed to identify the preferred product characteristics during in-process, release and stability evaluation of the vaccine targets and to ensure a reproducible safe product for pre-clinical and clinical use (Figure 1).

The success of RNA vaccines as defined by safety, immunogenicity, and efficacy will depend primarily on the quality of the transcript [6,12,13,14]. Quality attributes include, but are not limited to stability, integrity, identity, purity, and homogeneity. For example, cap structures at the 5′ end of the RNA enhance stability and promote translation, thus maximizing not only the lifetime of the transcript but also the resulting dose of the encoded target vaccine antigen [15]. Similarly, untranslated regions at the 5′ and 3′ ends [16,17,18,19] of intact transcripts are essential to maximizing translation. Known inhibitors of translation include process-derived double-stranded RNA species, aborted transcripts, as well as other degradants and contaminants. All these can trigger an innate inflammatory response, activate RNA sensors or promote RNA interference [20].

In this review, we provide a summary of the preferred quality control assays currently used to characterize mRNA vaccine antigen candidates at the biophysical, biochemical, and functional level. Even though most of these assays are widely used and well-established in the research laboratory, further optimization, qualification, and eventual validation will be needed to transition them for use during manufacturing campaigns. In fact, so far, no regulatory guidelines specific to mRNA vaccines quality control have been provided [21], although high-level guidance documents on the quality of biological therapeutics are certainly still useful. For example, just like other macromolecules, it may be prudent to evaluate RNA transcripts in terms of color and appearance, identity and integrity, concentration, potency, product- and process-related impurities, sterility, endotoxin levels, physicochemical properties such as pH, and osmolality, and particle size distribution, if the transcript is formulated with particulate excipients [21,22]. Moreover, we review various in vitro functional cell-based assays that have been employed to facilitate the early assessment of the biological activity of these molecules, including the predictive immune response triggered in their respective host. This allows equally rapid screening of immunogenicity, efficacy, and safety without the need to conduct often lengthy and costly in vivo experiments.

## 2. Characterizing DNA Templates and RNA Transcripts

### 2.1. Sequencing of the DNA Templates

Messenger RNA vaccines can be designed with great customization. For example, rare codons can be replaced to increase translation, and undesired secondary structures can be removed to avoid an undesired innate immune response [9]. DNA templates from which RNA transcripts are typically derived, e.g., plasmids, DNA duplexes, and PCR products, should be sequenced for accuracy since direct RNA sequencing has limited read length at present. In theory, mRNA transcripts can also be converted to complementary DNA that can then be sequenced, but reverse transcription in vitro suffers from several issues, including frequent reaction failures [22].

### 2.2. Removal of DNA Template and RNA Purification

Following production, RNA transcripts are digested with DNAse I to degrade DNA templates, and then purified to remove DNA degradants, reaction components, and other contaminants. Purification method include commerical spin columns, LiCl precipitation, phenol-chloroform extraction, and chromatography [23,24,25].

### 2.3. UV Spectroscopy

The most straightforward method to characterize RNA transcripts prima facie is UV spectroscopy [26,27]. For example, RNA can be quantified based on the consensus that a sample containing 40 µg/mL RNA typically has an absorbance of 1 at 260 nm. Measurement of UV absorbance does not require additional sample processing, except perhaps dilution or concentration to a suitable concentration if the absorbance falls outside the dynamic range of the spectrophotometer. UV absorbance is typically measured on instruments such as the NanoDrop® (Thermo Scientific, Waltham, MA, USA) and NanoVue™ (GE Healthcare, Chicago, IL, USA), which require only 0.5–2 µL of sample to quantify 2–2000 ng/µL RNA.

Care should be taken to measure absorbances against appropriate blanks, since many common biochemical reagents absorb UV light strongly, especially at shorter wavelengths, including buffers such as N-(2-acetamido)iminodiacetic acid. Indeed, non-specificity is a critical issue in UV spectroscopy, as for example, RNA degradation is undetectable by UV spectroscopy. Moreover, residual plasmids and nucleotides, if present, will also absorb UV light at 260 nm. DNAse I treatment is regularly employed to remove deoxyribonucleic acids from RNA preparation, yet the efficacy of the reaction needs to be confirmed. Furthermore, residual enzymes from transcription reactions have absorbance maxima at 280 nm, and other potential contaminants such as guanidine thiocyanate, a common component in commercial nucleic acid purification kits have absorption maxima in the UV range as well. Accordingly, A260/A280 and A260/A230 ratios are often superior indicators of purity, with highly-quality RNA producing ratios of 1.8–2.2 and >1.7, respectively [28]. Of note, the A260/A280 ratio is pH-dependent, so a sample to be measured should be buffered at slightly alkaline conditions (pH 7–8) to increase robustness, even though the product may not necessarily be stored in such buffer. Additional light-scattering contaminants may also be detected at 320 nm, although this can be treated as background and subtracted from other readings.

### 2.4. Fluorescence-Based Assays

The fluorescence of certain dyes, when bound to nucleic acids [17], can be exploited to quantify RNA, typically against reference standards of known concentrations. Dye-based assays are relatively fast, high-throughput, and extremely sensitive. For example, the QuantiFluor™ RNA System (Promega, Madison, WI, USA) can detect as little as 1 pg/µL, although, conversely, it can only quantitate up to 2.5 ng/µL, which is just above the lower limit of detection for Nanodrop instruments. Importantly, some dyes preferentially bind specific forms of nucleic acids, although most are not discriminating. For instance, the Quant-iT™ RNA Assay (Thermo Fisher, Waltham, MA, USA) is based on an RNA-specific dye, which, while less sensitive, might prove more useful if DNA contamination is suspected. Total nucleic acids can be measured by UV absorbance as previously described and then corrected for DNA contamination using Hoechst 33258 fluorescent dye [29,30]. While we understand that these types of controls for possible contaminants might not be needed in a research laboratory that relies on DNAse I treatment, we expect that they will be required in a regulated environment. As with UV spectroscopy, dye-based assays do not measure purity or integrity, although other dyes may be used in conjunction to quantify contaminating proteins. Generally, all dyes require the generation of an appropriate standard curve to allow accurate quantification and they all are potentially genotoxic [31], and thus require proper handling and disposal.

### 2.5. Electrophoresis on Agarose and Acrylamide Gels

Following separation by molecular mass on agarose or acrylamide gels, nucleic acids can be visualized using fluorescent dyes such as ethidium bromide, SYBR Green or SYBR Gold (Life Technologies, Carlsbad, CA, USA) [32,33,34], and then sized or quantified by densitometry using known RNA standards, as demonstrated for PhI p5, an allergen used to prevent allergic reactions to grass pollen [35]. The sharpness of the band is also sometimes considered an indicator of quality [23], as described for mRNA vaccines against influenza [36]. As with dye-based assays in solution, this method requires proper handling and disposal of potentially hazardous reagents [37], as well as capital equipment such as electrophoresis tanks, high-resolution scanners, and image-analysis software. Since this method is labor-intensive, integrated, miniaturized, microfluidic devices such as the 2100 Bioanalyzer (Agilent Technologies, Santa Clara, CA, USA) and Caliper Life Sciences’ GX II ((Waltham, MA, USA) may be used to reduce handling and increase throughput [38,39].

While gel-based methods are less sensitive, some specificity is achieved since the target RNA can potentially be separated from residual plasmid DNA or degradants. Nevertheless, contaminating proteins would still have to be quantified separately by other means. Importantly, gel-based methods can be used to measure RNA size and integrity. For instance, the 2100 Bioanalyzer outputs an RNA Integrity Number between 0 and 10, 10 representing RNA of high quality [38].

### 2.6. Reverse Transcriptase qPCR

Although not typically employed for this purpose, reverse transcriptase qPCR can also be used to quantify or characterize RNA transcripts produced in vitro. Messenger RNA transcripts are first reverse-transcribed to cDNA and then quantified by standard qPCR using DNA-binding fluorescent dyes or fluorescently labeled DNA probes or primers. As before, standards of known concentration can be assayed at the same time to measure the amount of RNA in each sample. If suitable primers can be designed, then the target RNA can also be directly quantified, as well as any residual plasmid. Primers that generate amplicons of various lengths can be used to determine RNA integrity since larger amplicons become consistently more difficult to amplify as RNA degrades. RT-qPCR is fast, high-throughput, and extremely sensitive, although it requires advanced training, capital equipment, and expensive reagents and consumables.

### 2.7. Western Blot for dsRNA

As double-stranded RNA is strongly immunogenic, its detection in and removal from RNA vaccines is critical, especially if RNA transcripts were produced in vitro [25]. These species can be detected by slot blot using monoclonal antibodies [25]. If necessary, double-stranded RNA can be degraded using RNAse III and the remnants purified away using standard methods [40].

### 2.8. Analysis of Capping

Capping at the 5′ end is a hallmark of eukaryotic and viral messenger RNA. Since capping affects stability and translation, proper quality control should include the quantitative assessment of capped and uncapped transcripts. In one method, a biotinylated tag is annealed to the 5′ end of the transcript, which is then cleaved via DNA 3′ end-directed cleavage by RNAse H. Subsequently, the hybrid fragment is isolated on streptavidin-coated magnetic beads, dissociated, and the untagged strand is analyzed by liquid chromatography-mass spectrometry (LC-MS). This method was reported to detect 0.5–25% uncapped RNA in 100 pmol of capped RNA [41]. If the residual fragments are sufficiently small to resolve by HPLC or TLC, then these may also be used to separate capped and uncapped transcripts (Patent WO2014/152659Al). Alternatively, capping efficiency can be estimated by incorporating (α-^32^P) GTP during in vitro transcription [42], which, however, would render the final product radioactive and thus unsuitable for clinical application. While it is possible to run parallel but separate reactions for clinical application and analysis, such reactions are sensitive to errors and minute variations due to the reaction conditions. Therefore, another approach digests transcripts using XRN1, a 5′–3′ processive exoribonuclease that digests uncapped mRNAs but not capped mRNAs [43]. This method though requires prior treatment of the transcript with RNA 5′-pyrophosphohydrolase to remove pyrophosphate from the 5′ end of triphosphorylated RNA to leave a 5′ monophosphate RNA [44].

### 2.9. Chromatography

Messenger RNA transcripts can be quantified or characterized by chromatography, including size-exclusion chromatography [45,46,47], anion-exchange chromatography, affinity chromatography, and reversed-phase chromatography [20]. In particular, size exclusion chromatography enables isolation of monomeric RNA from oligomeric species that form due to canonical or noncanonical base pairing, as well as purification from enzymatic reactions, removal of residual plasmid, and recovery of refolded RNA from aggregates that form after long-term storage [46]. Unfortunately, these techniques are severely limited by resolution and may only be appropriate for sufficiently small, resolvable transcripts. Indeed, the apparent molecular weight of RNA molecules on size-exclusion columns are about 4–6× larger than expected because of extended structure, severely restricting the dynamic range of commercially available resins and columns [46]. For large transcripts, chromatography may be useful only as a qualitative check, i.e., a sharp peak vs a broad peak, or as a check to ensure that the product is free of residual contaminants from transcription (e.g., free nucleotides) or processing (e.g., components of purification kits).

### 2.10. Characterization of RNA Transcripts Following Formulation

Although naked RNA transcripts can be physically delivered into cells by electroporation, they are, instead, often formulated with excipients that confer additional functional properties. For example, most delivery vehicles are expected or designed to protect against RNAses, and such protection is evaluated by electrophoretic mobility shift assay [23]. Transcripts formulated in particulate vehicles can also be evaluated for particle size, polydispersity, and zeta potential [23,25,48].

## 3. Characterizing mRNA Encoded Translation Products

### 3.1. In vitro Translation

To ensure that the mRNA transcript is in-frame and contains all elements necessary for translation, it can be translated in vitro using cell-free extracts [49,50]. Although any cell-free extract can theoretically be used for this purpose, only a few cell-free systems are commercially available, generally derived from metabolically active cells such as rabbit reticulocytes, wheat germ, or Escherichia coli. These systems are crude extracts containing the translational machinery, including ribosomes, tRNAs, aminoacyl-tRNA synthetases, initiation, elongation and termination factors. In addition, these systems are often supplemented with amino acids, energy sources (ATP, GTP) or energy regenerating systems, as well as other co-factors (Mg^2+^, K^+^, etc.). We note that although commercial systems are expensive, individual reactions can be scaled down to as low as 1 µL in order to drastically reduce costs when screening multiple transcripts, although care should be taken to prevent evaporation, such as by using a heated a lid.

Products from in vitro translation can then be visualized using standard Western blot if appropriate antibodies are available. If necessary, tags can be engineered into the transcript to enable immunodetection. Alternatively, the in vitro translation reaction can be supplemented with fluorescently labeled amino acids [51] which are then incorporated into the newly synthesized proteins so they can be directly visualized by SDS-PAGE. Since several samples can be loaded per SDS-PAGE gel, this approach saves time and labor and avoids the need for antigen-specific antibodies.

### 3.2. Messenger RNA Evaluation Using Cell-Based Systems

The next step for a complete evaluation of mRNA vaccine antigen candidates is to test the transfection of the molecules into the target cell and confirm their suitability for effective translation. In mRNA vaccine development this in vitro testing offers advantages over in vivo studies; a larger number of variables can be investigated and controlled in each experiment and the potential toxicity of specific molecules can be detected in a specific, safe and efficient way. This can be particularly useful when multiple vaccine candidates are being screened before entering the essential in vivo testing for validation.

Transfection rates can be best measured by Flow Cytometry using fluorescently labeled nucleotides, incorporated into the transcription reaction. Single-cell arrays are also available to rapidly assess transfection in support of parallel screening of multiple mRNA candidates [52,53]. Translation rates can be examined in vitro, for example, using self-reporting molecules.

When mRNA molecules are then first examined in cells, the context of the test system needs to be considered, as some cell lines are easier to transfect than others and translation efficiencies may also differ based on the host system. For example, Andries et al. evaluated modified nucleosides such as pseudouridine, N1-methylpseudouridine (m1ψ), 5-methylcytosine/pseudouridine and 5-methylcytosine/N1-methylpseudouridine (m5C/m1ψ) for their effect on transfection rates using self-reporting luciferase encoding mRNA. With this approach, they not only found generally increased transfection rates with m5C/m1ψ but, equally important, they also identified a more than 20-fold different luciferase activity depending on the target cell line [54]. While in HeLa cells they measured a relative activity of 9 × 10^6^ RLUs (Relative Light Units), in keratinocytes they only observed 2.5 × 10^6^ RLUs. The authors further observed that the cell line background influenced the impact of certain nucleoside modifications on translation efficiency; m5C/m1ψ had the biggest effect in all cell lines except C2C12, where actually m1ψ yielded the biggest benefit, further emphasizing the importance of considering what cell line is used in a particular experiment.

### 3.3. Evaluation using Professional Antigen Presenting Cells

To date the most commonly used cell type to test mRNA vaccines are dendritic cells (DCs), antigen-presenting cells (APC) that can stimulate (capture, process, and present antigens) naïve T cells, resulting in cell-mediated immunity. Upon pathogen recognition, the innate and adaptive immune responses can upregulate components of the Major Histocompatibility Complex (MHC) molecules as well as co-stimulatory receptors. In addition, DCs can promote self-tolerance by secreting tolerogenic cytokines that induce the differentiation of regulatory T cells. As a result, there is considerable interest in DCs as potential therapeutic targets [55]. The challenge in the use of the DCs, however, is the technical difficulty of isolating these cells ex vivo as well as their general vulnerability [56].

There are two different stages in the life cycle of DCs: Immature DCs (imDC), found in the periphery where they patrol pathogens, apoptotic cells, and antigens from the outside and process them with great efficiency. However, their ability to present antigens and stimulate T cells is limited. They are also characterized by low cytokine production and low expression of MHC molecules. Mature DCs (mDCs), though, are characterized by a high expression of MHC components, as well as co-stimulatory and adhesion molecules. Furthermore, mDCs are efficient in antigen-presentation to T cells (CD4^+^ or CD8^+^ T cells) and have the ability to migrate to the lymph nodes to present antigens to T cells. In the lymphoid organs and other tissues, the conventional tissue-resident DCs (cDC) act as sentinels for antigen capture and presentation in situ. Other inflammatory DCs may differentiate from blood-derived monocytes and infiltrate secondary organs and tissue during infection as part of the inflammatory response [56]. While some studies report equal transfection rates in different DCs [57], there is evidence that the transfection efficiency for mRNA is lower for mDCs than it is for imDCs [58].

**Figure 2:**
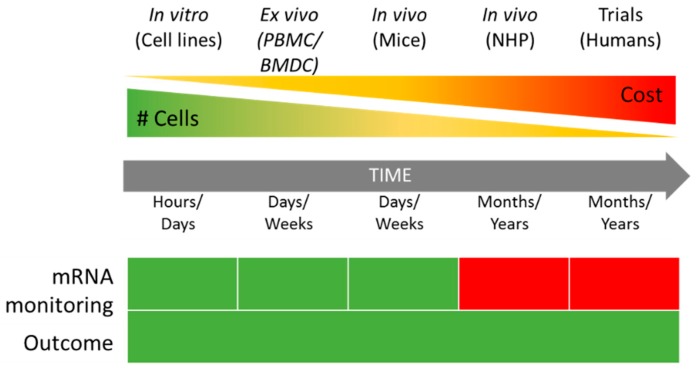
Comparison between in vitro, ex vivo, and in vivo approaches to mRNA vaccine evaluation in terms of cost and time. mRNA monitoring: Green—mRNA transfection and translation can be monitored by flow cytometry and western blotting. Red—no easy tracking possible. #—Number of cells available. PBMC—Peripheral Blood Monocytes Cells, BMDC—Bone-Marrow Derived Cells, NHP—Non-human Primates.

Compared to bone-marrow-derived cells (BMDCs) or peripheral blood mononuclear cells (PBMCs), immortalized cell lines save both time and costs (Figure 2) as they reduce the need for the repetitive sacrifice of mice or frequent sampling of human blood [56].

Transfection efficiency of mRNA construct has therefore primarily been tested in DC2.4 cells. For example, this immortalized cell line was used to test mRNA delivery using self-assembled cationic nanomicelles. Uptake was evaluated using fluorescently labeled mRNA in conjunction with various delivery platforms and quantified using flow cytometry. The expression of the mRNA was verified using EGFP (enhanced Green Fluorescent Protein) encoding mRNA, and the resulting protein was again quantified by flow cytometry. Overall, the authors were able to use this approach to demonstrate the benefit of their nanoparticles for the translation of the target RNA [59]. To further test the immunogenicity of the mRNA then, the authors used a product (Prostate Specific Antigen, PSA) specific assay that involved the maturation of murine bone marrow-derived dendritic cells (BMDCs), as well as antigen-specific antibodies.

In another example of the use of DC2.4 cells to quality control therapeutic mRNA candidates, Haifa Shen’s laboratory is studying cellular uptake of their anti-cancer therapeutics. Efficient internalization was initially tested using GFP (Green Fluorescent Protein) as a self-reporting marker, combined with Flow Cytometry to quantify the number of GFP-positive cells. Without having to go into animals, the authors were further able to test the uptake of fluorescently labeled mRNA in the presence of various uptake inhibitors, to better understand the endocytic pathway of how their particular mRNA vaccine enters the dendritic cells [60].

Aside from Flow Cytometry, fluorescence Microscopy has proven to be a valuable tool to monitor transfection and the time course of antigen mRNA expression and to evaluate different delivery systems in vitro [61,62,63,64,65,66]. More recently, live cell video microscopy has also been used to track the uptake of fluorescently labeled mRNA cell line [67,68].

Other studies to understand the innate immune response produced by mRNA constructs have been conducted using BMDCs. When DCs from BALB/c mice were transfected with mRNA (Luciferase and EGFP), with and without modified nucleosides, a reduction in the innate immune response was associated with the modification likely also responsible for an increase in translation. The same study used mRNA purified by HPLC, and conclude that the purification increased the translation while also controlling the innate immune response [20]. Studies of human DCs ex vivo though have been constrained by difficulties; therefore, the majority of studies have been carried out in other systems. These have proven to be extremely powerful, generating a large body of fundamental data and describing numerous aspects of DC functions alongside a body of translational research using DCs for the cellular adoptive immunotherapy of infection and cancer [69]. One of the greatest problems of the ex vivo model is genetic variability, as evidenced by the fact that almost all cancer therapies are personalized. In addition, to obtain 10 million DCs, 100 ml of fresh blood are needed. Initial transfection studies with mRNA have been performed with PBMCs, but additional work is pending [70]. Van Gulk et al., used PBMCs from patients infected with HIV to obtain DCs and transfect them with mRNA encoding the viral gag and env proteins as novel immunotherapies against the virus [71]. Moreover, Peripheral Blood Mononuclear Cells (PBMCs) and BMDCs were used to test an mRNA vaccine against ZIKA virus to verify the translation of an experimental mRNA vaccine before transition to mouse and macaques models [72].

### 3.4. Evaluation Using Alternative Cell Lines

Alternative types of cells have also been used to evaluate and characterize mRNA vaccine candidates against specific pathogens before in vivo evaluation. The PR8HA antigen, for instance, was tested in HeLa cells as an mRNA vaccine candidate against Influenza A. Translation efficiency invitro of the PR8HA mRNA was measured by flow cytometry, as well as intracellular (99.6%) and surface (42.7%) staining. After the high translation efficiencies observed in the cell culture model, testing was moved to the mouse model, where 100% survival upon lethal challenge was observed [73]. In 2013, Hekel et al., used Self-Amplifying mRNA (SAM) to target the influenza virus H7N9. Within only 5 days of the publication of the viral sequence, the authors generated an mRNA vaccine candidate, emphasizing the importance of the mRNA vaccines in outbreaks of new pathogens. To quality control their vaccine candidates, SAM (H1) and SAM (H7), HeLa cells were transfected, and western blots were used to confirm the translation of the antigen in the cells. In immunogenicity studies HA-specific antibodies were quantified in serum 14 days after the first and second vaccinations, eliciting hemagglutination inhibition and neutralization titers against the virus [74]. More recently, Magini et al., used BHK-1 cells to determine the translation efficiency of two other SAM vaccine candidates against influenza, the influenza Nucleoprotein (NP) and the Matrix protein 1 antigen (MP1). When mRNA efficacy was evaluated by flow analysis, cells were found to be transfected with both mRNAs at rates of 77 and 85.8%, respectively. For a bicistronic NP-MP1 SAM RNA, 79.7% of the cells were found to be transfected. As predicted by these in vitro studies, a significant increase in the survival of vaccinated mice was then observed after challenge with influenza virus [75].

In another example, mRNA vaccines against the rabies virus encode the surface glycoprotein RABV-G. Before being used in animals, the translation of the RABV-G was tested in HeLa cells by flow cytometry, where 99.6% of the cells were positive for the antigen. Subsequently, the survival of challenged and vaccinated mice was 100% against lethal intracerebral rabies challenge infection [76]. For the vaccine against the Zika virus, the prM and E genes from an Asian Zika virus strain (Micronesia 2007) were used for vaccine development. Here, HeLa and HEK-293 cells were transfected with prM-E mRNA which resulted in efficient expression and secretion of antigen, as demonstrated by Western blot [77]. Another SAM RNA vaccine against streptococcal infections was first tested in BHK-V cells, which were transfected with BP-2a-, LS-BP-2a- and SLOdm-encoding SAM mRNAs and then validated by Western blot analysis to demonstrate the expression of the target proteins [78]. After successful confirmation of transfection and translation in vitro, the vaccine was shown capable of protecting mice against Group A *Streptococcus pyogenes* (GAS) and Group B *Streptococcus agalactiae* (GBS).

## 4. Summary

Messenger RNA as a novel vaccine platform holds great promise, but, as all biologics, it will require careful characterization in order to ensure quality, safety, and efficacy. We have tried to compile an array of published biophysical and biochemical tools as well as cellular immunology tools that are necessary to evaluate the quality characteristics of vaccine antigen candidates before advancing them into more costly pre-clinical testing. With this toolbox of assays in place, mRNA vaccine antigen candidates can swiftly be advanced towards in-vivo testing. It is noted though that the efficacy of an mRNA in vivo will likely strongly depend on its formulation and the delivery platform. Even at the level of cellular testing, multiple approaches are still being investigated (Table 1) and while, for example, electroporation and lipofectamine may yield satisfactory transfection rates in vitro, alternative compositions, as reviewed by others [3,79,80], may be needed in vivo. With these tools aligned though, there is high potential for this platform to greatly expand the options in accelerating vaccine development.

## Figures and Tables

**Table 1:** Delivery systems for the evaluation of the mRNA constructs.

Cell	Delivery System		Ref.
	In vitro	In vivo	
A549, BJ, C2C12, HeLa, and keratinocytes	Lipofectamine 2000	Lipofectamine 2000	[54]
HeLa	Lipofectamine 2000	RNActive, CureVAC	[73]
BHK	Lipofectamine	Lipid nanoparticle	[74]
BHK-21	Electroporation Lipofectamine 2000	Lipid nanoparticle	[75]
HeLa	Protamine-RNA Complex	Protamine-RNA Complex	[76]
BHK-V	Electroporation	Cation-Nano-Emulsions	[78]
DC 2.4 * BMDC *	PEI nanoparticles	N/A	[59]
DC 2.4 *	Lipopolyplex	N/A	[60]
BMDC *	Lipofectine and TransIT	N/A	[20]
PBMC *	Electroporation	N/A	[71]
HEK-293T PBMC * and BMDC *	TransIT	Lipid nanoparticle	[72]

* Dendritic Cells (APC). N/A—No data available. BHK—Baby Hamster Kidney fibroblasts; BMDC—Bone-Marrow-Derived Cells; PBMC—peripheral blood mononuclear cells; HEK-293T—Human embryonic kidney 293 cells; PEI—polyethylenimine.
